# Interactions between HIV infection and chronic obstructive pulmonary disease: Clinical and epidemiological aspects

**DOI:** 10.1186/1465-9921-12-117

**Published:** 2011-09-01

**Authors:** Christine Raynaud, Nicolas Roche, Christos Chouaid

**Affiliations:** 1 Service de pneumologie, Centre Hospitalier Victor Dupouy, 69 rue du L.C. Prud'hon, 95100 Argenteuil, France; 2 Service de pneumologie et réanimation, Hôtel Dieu, AP-HP, Université Paris Descartes, 1 Place du parvis de Notre Dame, 75004 Paris, France; 3 Service de pneumologie, Hôpital Saint Antoine, AP-HP, Université Pierre et Marie Curie, 184 rue du Fbg St Antoine, 75012 Paris, France

## Abstract

**Introduction:**

An association between HIV infection and chronic obstructive pulmonary disease (COPD) has been observed in several studies.

**Objective and methods:**

we conducted a review of the literature linking HIV infection to COPD, focusing on clinical and epidemiological data published before and during widespread highly active antiretroviral therapy (HAART).

**Results:**

Interactions between HIV infection and COPD appear to be influenced by multiple factors. In particular, the bronchopulmonary tract can be damaged by HIV infection, the immunodeficiency it induces, and the resulting increase in the risk of pulmonary infections. In addition, the prevalence of smoking and intravenous drug use is higher in HIV-infected populations, also increasing the risk of COPD. Before the advent of HAART, respiratory tract infections probably played a major role. Since the late 1990s and the widespread use of HAART, the frequency of opportunistic infections has fallen but new complications have emerged as life expectancy has increased.

**Conclusion:**

given the high prevalence of smoking among HIV-infected patients, COPD may contribute significantly to morbidity and mortality in this setting.

## Introduction

Chronic obstructive pulmonary disease (COPD) is defined by slowly progressive, incompletely reversible chronic airflow obstruction. Smoking is the main risk factor but the mechanisms linking smoking to COPD are not completely clear, although they are known to involve inflammation, oxidative stress, proteolytic injury and both innate and acquired immunity [1,2]. Active smoking and frequent exacerbations (often related to lower respiratory tract viral or bacterial colonization and infection) are associated with COPD progression, but other factors may be involved.

The possibility of an increased susceptibility of HIV-infected patients to COPD has been raised in recent years [3-5]. Both HIV infection *per se *and the resulting immune response have been implicated. Moreover, HIV-infected patients appear to be at a particular risk of developing COPD, owing to their high prevalence of smoking, recurrent pulmonary infections (including opportunistic infections), frequent drug use, and often precarious socio-economic status [5]. However, these features also represent confounding factors in analyses of the possible association between COPD and HIV infection [5].

The impact of highly active antiretroviral therapy (HAART) adds to the complexity of this issue. HAART consists of a combination of at least three antiretroviral drugs taken simultaneously and regularly in order to achieve a maximal reduction in viral load [6]. During the pre-HAART period, mortality among HIV-infected patients was mainly due to opportunistic infections. Since the end of the 1990s and widespread HAART use, life expectancy of HIV-infected subjects has increased and new complications have emerged [7,8]. The high prevalence of smoking in this now aging HIV-infected population means that COPD may be a significant source of morbidity and mortality [4,9].

The aim of this literature review is to examine interactions between HIV infection and COPD, focusing on clinical and epidemiological data published in the pre-HAART and HAART eras.

### COPD-HIV interaction in the pre-HAART period (Table 1)

**Table 1:** Main published studies on COPD-HIV interaction in the pre-HAART era

Year of publication	First author Reference	Study period	Type of study	Focus	Number of patients	Main findings
1993	Moscato G [12]	NR	"One day" Case-control	Bronchial hyper-responsiveness	25 (25 controls)	Prevalence of bronchial hyper-responsiveness identical in the two populations

1997	Wallace JM [11]	1988-1994	Cohort Case-control	Bronchial hyper-responsiveness	62 (62 controls)	Prevalence of bronchial hyper-responsiveness identical in the two populations

2001	Poirier CD [10]	1995-1996	prospective Case-control	Bronchial hyper-responsiveness	248 (236 controls)	Prevalence of bronchial hyper-responsiveness identical in the two populations Among smokers, bronchial hyper-responsiveness more frequent in HIV-infected men than in controls

2003	Diaz PT [13]	1993-1998	Cohort Case-control	Chronic bronchitis	327 (52 controls)	Chronic bronchitis more frequent in HIV-infected patients than in controls (26.9% versus 13.5%, p < 0.05)

1998	Shaw RJ [16]	NR	Prospective? descriptive	Airway obstruction	34	Lung infections (PCP, bacterial pneumonia) associated with lower FEV_1 _and peak flow rates

1988	O'Donnell CR [14]	1983-1986	Retrospective?	Airway obstruction	99 (AIDS)	Estimated prevalence of lower forced expiratory. flow rates: 33%

1999	Gelman M [15]	NR	Prospective Case-control	Air trapping/CT	48 (11 controls)	Degree of air trapping correlated with duration of HIV infection

2000	Hnizdo E [18]	1995-1996	Cohort Case-control	Impairment of lung function and tuberculosis	305 (1038 controls)	Functional respiratory decline due to tuberculosis not aggravated by HIV co infection

2000	Morris AM [17]	1988-1994	Cohort descriptive	Airway obstruction	141	Acceleration of decline in FEV_1_, FVC and FEV_1_/FVC, for several months after acute episode

1989	Kuhlman JE [21]	NR	Retrospective descriptive	Emphysema CT findings	55	CT signs of emphysema, bullous lesions and cysts in 42% of cases

1996	Guillemi SA [22]	NR	Prospective descriptive	Emphysema CT findings	32	CT signs of emphysema in 31% of cases

1999	Diaz PT [20]	NR	Prospective Case-control	Emphysema	96 (30 controls)	CT signs of emphysema in 50% of cases in patients with reduced D_L, CO_

2000	Diaz PT [23]	1994-1997	Prospective Case-control	Emphysema	114 (44 controls)	Increased incidence of emphysema in the HIV-infected population (15% versus 2% in controls (p = 0.025)).

1993	Nieman RB [25]	1986-1991	Cohort descriptive	T_L, CO_	84 (AIDS)	Decline in T_L, CO _significantly associated with more rapid progression to AIDS

1993	Mitchell DM [27]	NR	Cohort descriptive	D_L, CO_	474	D_L, CO _decline in case of acute respiratory disease. Decline not specific for PCP diagnosis.

1995	Rosen MJ [29]	1988-1994	Prospective Case-control	D_L, CO_	1127 (167 controls)	CD4 < 200/mm3, ethnic origin, smoking, IV drug use associated with D_L, CO _decline

Several studies have examined the link between HIV infection and bronchial hyper-responsiveness, a possible early sign of airway damage. The prevalence of bronchial hyper-responsiveness is not significantly influenced by HIV infection [10-12]. In contrast, the prevalence of bronchial hyper-responsiveness seems to be higher among HIV-infected male smokers (enrolled between 1995 and 1996) than in their seronegative counterparts, suggesting that HIV-infected men may be more sensitive to the consequences of smoking [10].

Another study (conducted between 1993 and 1998) showed a higher frequency of chronic bronchitis (defined as near-daily expectoration for three months or more per year) in HIV-infected patients than in a control group (26.9% versus 13.5%, p < 0.05), despite a similar proportion of current smokers (about 50%) and a trend towards lower cumulative smoking (12.2 ± 0.9 vs 17.2 ± 3.2 pack-years, p = 0.13) [13]. D_L, CO _was lower in these patients, and a trend towards lower FEV1 was reported (91.8 ± 0.7% vs 94.9 ± 1.8%, p = 0.12). Among HIV-infected subjects, respiratory symptoms were more frequent in smokers, IV drug users, patients with a history of asthma. In a population of AIDS patients undergoing routine pulmonary function tests (PFT), the estimated prevalence of reduced forced expiratory flow rates was 33% [14].

Airways involvement has also been suggested in a CT-scan study of 59 subjects, which found evidence of expiratory air trapping in 30/48 HIV-infected patients versus 3/11 HIV-seronegative subjects [15]. Air trapping was associated with lower FEV1 and D_L, CO_. Subjects with air trapping tended to have lower CD4 counts, but the difference was far from significant (p = 0.40). Both acute and chronic respiratory tract infections play a noteworthy role in bronchial obstruction [16]. Pulmonary infections (*Pneumocystis carinii *pneumonia, bacterial pneumonia) are associated with durable changes in respiratory function in HIV-infected patients. The Pulmonary Complications of HIV Infection Study Group performed PFT every 3 to 12 months in 1149 HIV-infected patients between 1988 and 1994 [17]. In this cohort, 141 patients having had *Pneumocystis carinii *pneumonia (PCP) or bacterial pneumonia were followed-up, and a permanent decrease in FEV_1_, FVC and FEV_1_/FVC ratio was observed, lasting several months after resolution of the acute episode. In contrast, the decline in respiratory function following pulmonary tuberculosis was not accelerated by HIV co-infection [18].

HIV infection appears to be associated with parenchymal involvement, and especially emphysema. In 1992, Diaz *et al*. [19] reported the cases of four HIV-infected patients (including 3 smokers) with no history of pulmonary infection, who presented with dyspnea and were diagnosed with "emphysema-like syndrome". Pulmonary function tests showed air trapping, hyperinflation and reduction in CO diffusing capacity despite minimal airflow obstruction. Computed tomographic scans were performed in three of these patients and revealed bullous changes, pointing to a possible link between HIV infection and emphysematous pulmonary tissue destruction [19]. The same authors, in a population with no history of pulmonary infection but with diminished diffusing capacity of the lung for carbon monoxide (D_L, CO_) (< 72% predicted), found CT evidence of emphysema in 50% of cases [20]. In another study of 55 patients with AIDS, radiological evidence of emphysema, bullous lesions or cysts was found in 42% of cases and was associated with a history of pulmonary infection in 70% of cases [21]. During routine CT studies prior to initiation of primary PCP prophylaxis, signs of emphysema were found in 31% of cases [22]. A study published in 2000 compared lung function in 114 HIV-infected patients (enrolled between 1994 and 1997) and 44 uninfected controls matched for age, sex and smoking status [23]. The authors confirmed the higher incidence of emphysema associated with HIV infection (15% versus 2%, p = 0.025). The difference seemed to be even more marked among smokers. However, it should be noted that fewer than 10% of these patients, studied between 1994 and 1997, were receiving HAART.

Several studies have shown abnormal CO diffusion in HIV-infected patients, but the exact origin of this abnormality has not been determined. Diaz *et al*.[20] found that diffusion impairment in HIV-infected patients was associated with loss of capillary blood volume (Vc) and not with an alteration of the membrane component (Dm). This suggests that the decline in T_L, CO _is not related to infiltration of the alveolar space or interstitium but rather to vascular abnormalities or emphysema. Reduced carbon monoxide transfer factor (T_L, CO_) values were observed outside the context of respiratory tract disease [24,25], and also during the acute phase of PCP [16,26-28]. The reduction in D_L, CO _appears to be significantly more marked in patients with CD4 cell counts below 200/mm3 [29] and to correlate with more rapid progression to AIDS [25]. It may be linked to pulmonary inflammation related to immunodeficiency. In some patients, the reduction in D_L, CO _can also be related to undiagnosed HIV-related pulmonary hypertension [30].

Before the widespread use of HAART, several observations showed connections between COPD and HIV. Particular, emphysematous and bullous diseases have been described in young patients. Respiratory tract infections (opportunistic or not) were very common during this period and may had played a major role in the pathogenesis of COPD. As morbidity and mortality were essentially due to AIDS at that time, the impact of obstructive lung disease was not well known.

### COPD-HIV interaction in the HAART era (Table 2)

**Table 2:** Main published studies on COPD-HIV interaction in the HAART era

Year of publication	First Author reference	Study period	Type of study	Focus	Number of patients	Main findings
2005	Crothers K [32]	1999-2000	Observational study prospective cohort	Respiratory symptoms	867	Smoking associated with increase in respiratory symptoms; cough and dyspnea found in 44% of smokers and 25% of non smokers

2006	Crothers K [31]	2001-2002	Observational study Prospective Case-control Cohort	COPD (self-assessment and coding data)	1014 (713 controls)	Self-assessment: prevalence of COPD significantly higher in HIV-infected patients (15% vs 12%, p = 0.04); HIV infection = independent risk factor for COPD

2009	George MP [33]	2003-2004	Observational study Prospective	Respiratory symptoms. airway obstruction	234	Prevalence of airway obstruction: 6.8%. Age, pack-years, history of bacterial pneumonia and HAART = independent risk factors for airway obstruction

2009	Morris A [43]	NR	Observational study Prospective	*Pneumocystis *colonization and airway obstruction	42	Colonization by *Pneumocystis jirovecii *(26% of cases) associated with increase in airway obstruction and sputum metalloprotease (MMP 12) levels

2010	Drummond MB [36]	1988-?	Observational study Prospective Cohort Case-control	Respiratory symptoms. Airway obstruction	288 (686 controls)	Prevalence of airway obstruction: 15.5%. No influence of HIV status

2010	Cui Q [35]	NR	Observational study Prospective	Respiratory symptoms. Airway obstruction	119	No acceleration of FEV_1 _decline relative to published data for general population

2010	Gingo RM [34]	2007-2009	Cross-sectional analysis	Airway obstruction	167	64% of patients had impaired diffusion. 21% of patients had irreversible airway obstruction. Irreversible airway obstruction was independently associated with HAART, pack-years smoked and intravenous drug use.

2011	Crothers K [37]	1999-2007	Observational study prospective Cohort Case-control	Coding data	3707 (9980 controls)	HIV-infected patients more likely to have diagnoses of COPD (20.3 per 1000 person-years versus 17.5 per 1000 person-years - p < 0.001).

The impact of HAART on respiratory disorders can be envisaged from different angles. On the one hand, assuming that HIV has inherent pathogenicity for the respiratory tract, HAART could prevent COPD by inhibiting viral replication. It could also prevent COPD by reducing the frequency of opportunistic infections and their long-term consequences.

On the other hand, the increase in life expectancy among HIV-infected patients, who have a high prevalence of smoking, could lead to an increase in the incidence of COPD. Nevertheless, no increase in the prevalence of airway obstruction has been observed among HIV-infected patients in recent years.

**Figure 1:**
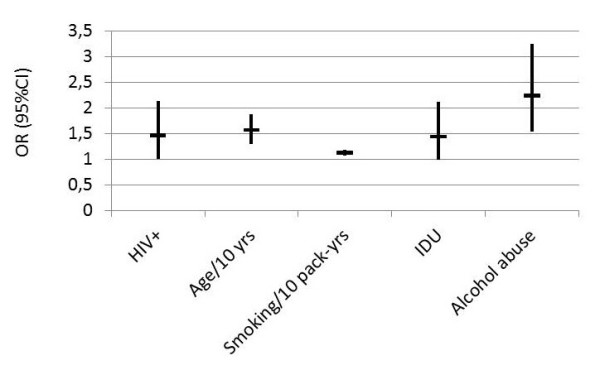
**Predictors of COPD (OR with 95%CI) for some of the risk factors identified (using ICD-9 codes) in multivariate analysis as independently associated with a diagnosis of chronic airflow obstruction, among 1014 HIV-positive and 713 HIV-negative US veterans **[31].

In 2006, a prospective observational study comparing 1014 HIV-infected patients with 713 uninfected controls enrolled between 2001 and 2002 was published [31]. The prevalence of COPD was determined from coding data (International Classification of Diseases, ninth revision (ICD-9)) and also from a self-assessment in response to the question: "Has a doctor ever told you that you had a chronic lung disease (emphysema, asthma, chronic bronchitis, or chronic obstructive lung disease)?" In univariate analysis, based on the coding data, the prevalence of COPD was identical in the two populations; in contrast, the self-assessment suggested that the prevalence of COPD could be higher in the HIV-infected patients (15% vs 12%, p = 0.04). Indeed, multivariate analysis identified HIV infection as an independent risk factor for COPD (Figure 1) [31]. The lack of spirometric measurements and the failure to take into account environmental and occupational exposures are the main limitations of this study. Another study of a cohort of 867 HIV-infected patients showed that smoking was associated with an increase in respiratory symptoms; cough and dyspnea were found in 44% of smokers and 25% of non-smokers control [32].

**Figure 2:**
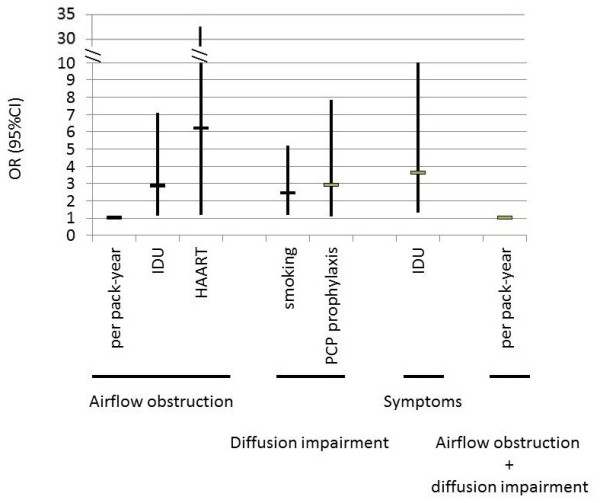
**Predictors of COPD (OR with 95%CI) for risk factors independently associated with airflow obstruction, diffusion impairment, both, and symptoms in a cohort of 167 HIV-infected subjects **[34]. Age/10 y: age in ten-year periods. Smoking/10 pack-years: smoking per ten pack-years.

A more recent prospective observational study examined the prevalence and risk factors of respiratory symptoms and airway obstruction in a population of 234 HIV-infected patients. The median CD4 count was 371 cells/mm^3 ^(interquartile range 3 - 1368). A majority of patients were on HAART (83%). The duration of disease was 8 years among HAART users and 10 years among patients not using HAART. The prevalence of airway obstruction was 6.8% [33]. One year later, in a cross-sectional study, 167 HIV-infected patients had PFT [34]. The median CD4 count was 479 cells/mm^3 ^(interquartile range 22 - 1390). A majority of patients were on HAART (80.7%). The median disease duration was 13 years (range 0.1-27). Irreversible airway obstruction was found in 21% of this population. Age, cumulative smoking history, a history of bacterial pneumonia, intravenous drug use and HAART were independent risk factors for bronchial obstruction (Figure 2) [33,34]. In contrast, a cross-sectional study establishing a link between cumulative smoking and FEV_1 _in 120 HIV-infected patients (-2.1% of FEV1 per 10 pack-years) found a similar loss of FEV_1 _in HIV-infected smokers by comparison with published data for smokers in the general population [35]. However, this was a cross-sectional study, excluding a formal assessment of rates of FEV1 decline. In a cohort of IV drug users, the prevalence of airway obstruction was 15.5% overall and did not differ according to HIV status [36]. In this study, 54.6% of patients reported HAART use (median CD4 count 322 cells/mm^3 ^(interquartile range 177 - 503)) and the others had a median CD4 count of 321 cells/mm^3 ^(interquartile range 178 - 497). HIV status was not associated with chronic cough and sputum production either, but HIV-positive individuals reported moderate-to-severe dyspnea (MRC dyspnea grade ≥ 2) more frequently (OR 1.50, 95%CI: 1.08-2.09). D_L, CO _and CT-scan were not available in this study, preventing from assessing the role of emphysema. Conversely, hemoglobin levels were slightly but significantly lower in HIV+ subjects, which could contribute to explain the higher frequency of dyspnea.

More recently, Crothers *et al*. assessed pulmonary diseases in a large cohort of 3707 HIV-infected patients (65% on HAART; median CD4 count 264 cells/mm^3 ^(interquartile range 108 - 151)) [37]. This cohort was demographically matched to 9980 HIV-uninfected patients. Pulmonary conditions were diagnosed based on ICD-9 codes (International Classification of Diseases, ninth revision). HIV infection was independently associated with a significantly higher risk of COPD (20.3 per 1000 person-years versus 17.5 per 1000 person-years; p < 0.001). Among HIV-infected subjects, the incidence of COPD was lower in patients on HAART (65% of the population studied) and in patients with baseline HIV RNA levels below 400 copies/ml [37].

### Factors underlying COPD-HIV interaction (Figure 3)

**Figure 3:**
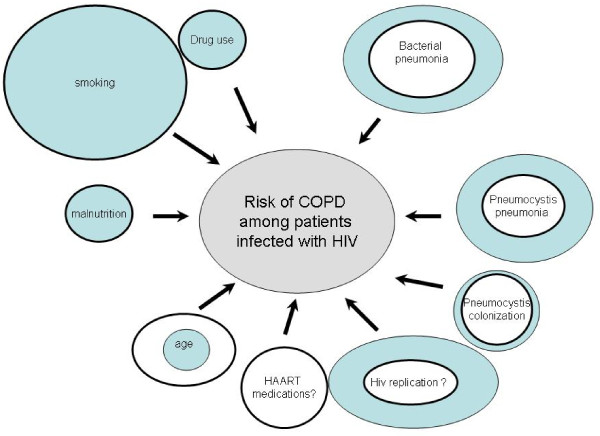
**Factors underlying COPD-HIV interaction and their evolutions between pre-HAART era (blue circles) and HAART era (white circles)**.

Several factors are involved in the COPD-HIV interaction. Some are directly linked to HIV infection, others relate to its complications (colonization by *Pneumocystis jirovecii, Pneumocystis jirovecii *pneumonia, bacterial pneumonia, etc.) and its treatment. Other risk factors such as smoking and IV drug use are also more frequent in the HIV-infected population.

A possible role of HIV itself in the onset of COPD is supported by data on the pathophysiology of COPD. HIV infection causes intense pulmonary infiltration by CD8^+ ^lymphocytes, which are known to be involved in the development of COPD [3]. In addition, gamma-interferon production is increased in the lungs of HIV-infected patients at different stages of the disease, including the asymptomatic phase [3]. However, the exact role of pulmonary effects of HIV infection in the pathogenesis of COPD remains to be determined. In addition, HIV transgene expression in rats and treatment of cell cultures with HIV-related proteins (gp120 and Tat) induce oxidative stress and reduce the expression of alveolar epithelial tight junctions, which may participate to explain lung function abnormalities in HIV-infected humans [38].

Recent data suggest that colonization by *Pneumocystis jirovecii *may also be involved in the development of COPD, by inducing an inflammatory reaction and stimulating the production of metalloproteases in the lung [39]. *Pneumocystis jirovecii *colonization correlates with the degree of bronchial obstruction, independently of smoking status, in HIV-seronegative COPD patients [39-41]. Pulmonary colonization by *Pneumocystis jirovecii *is frequent in HIV-infected patients, one study showing a prevalence of 46% in patients who died of causes other than PCP [42]. In a group of 42 HIV-infected patients with no signs of PCP, induced sputum analysis revealed *Pneumocystis jirovecii *colonization in 26% of cases. Bronchial colonization by *Pneumocystis jirovecii *was associated with an increase in bronchial obstruction (reduction in FEV_1 _and the FEV_1_/FVC ratio) and an increase in sputum metalloprotease (MMP 12) levels [43]. In those studies, the CD4 cell count was not related to the risk of *Pneumocystis *colonization [42,43]. In a primate model of AIDS, *P. jirovecii *colonization was associated to COPD-like abnormalities, *i.e*. an accelerated decline in lung function and radiological and histological features of emphysema [44]. Th2 cytokines were increased in the BAL fluid of these animals, in which the proportion of airways associated with bronchus-associated lymphoid tissue was increased.

HIV-infected patients are at an increased risk of bacterial pneumonia. This risk increases markedly when the CD4+ cell count falls below 200/mm3, and in case of IV drug use [45]. A recent study confirmed that a history of bacterial pneumonia is an independent risk factor for airway obstruction in the HIV-infected population [33]. One mechanism underlying the deleterious effect of bacterial pneumonia on lung function decline among HIV-infected patients could be an HIV-induced increase in lung oxidative and nitrosative response to endotoxins, as found in a transgenic mouse model [46].

There also seem to be links between HAART and airway obstruction [33,34,37]. The study by Crothers *et al*. [37] showed a protective effect of HAART on the development of COPD, but airflow obstruction was not specifically examined. Conversely, in two other studies, HAART use was independently associated with bronchial obstruction [33,34].

Several pathophysiological explanations have been forwarded. Direct effects of HAART on the lung could exist, similar to HAART-associated cardiovascular disease, metabolic syndrome and osteoporosis. The Immune Reconstitution Inflammatory Syndrome (IRIS), a well-documented side-effect of HAART, might also be involved in airway obstruction. The restoration of the immune system could also induce an inflammatory response to sub clinical infections, perpetuating lung damage. It has also been hypothesized that an autoimmune response could develop after initiating HAART [33,47].

The prevalence of airway obstruction in IV drug users appears to be high (15.5%) but does not seem to differ according to HIV status [36]. In a large population of IV drug users, a reduction in D_L, CO _was found in 42% of cases. This anomaly was usually isolated and was attributed to vascular phenomena (foreign particle emboli) [48]. Sherman *et al*. reported the cases of 6 patients who used methylphenidate (Ritalin^®^) and presented with airway obstruction and a reduction in their D_L, CO _values [49].

The prevalence of smoking among HIV-infected patients ranges from 40% to 70%, compared to about 25% in the general population in the United States [4,35,50-54]. In the pre-HAART period, the impact of smoking on mortality among HIV-infected patients was controversial, some studies showing no difference between smokers and non-smokers [55,56], while others [57] showed more rapid disease progression and a higher risk of death among smokers. Since the advent of HAART, smoking has been identified as a significant risk factor for mortality among HIV-infected patients [32,54], although the precise causes remain to be identified. Cardiovascular disorders and cancer probably also contribute to this higher mortality. More prolonged survival likely allows tobacco smoke to exert its deleterious effects and gives time for the corresponding diseases to become symptomatic. Interestingly, cannabis and tobacco smoke appear to produce synergistic effects on lung function, although the impact of cannabis alone is controversial [58]. This may be of particular relevance since (i) substance abuse is higher among HIV-infected subjects than in the general population [59] and (ii) medical marijuana has been legalized in some countries or states [60].

## Conclusion

The prevalence of COPD is higher in the HIV-infected population than in the general population. Before the HAART era, respiratory tract infections were a very important risk factor. The advent of HAART has led to changes in HIV-related pulmonary diseases and to longer survival. COPD is emerging as a new source of morbidity in HIV-infected patients, with a deleterious synergistic interaction between HIV, smoking and COPD. These interactions deserve a particular attention, and efforts must focus on smoking prevention, particularly in the HIV-infected population. In HIV-infected patients (smokers and non smokers), clinicians should be aware that chronic respiratory symptoms necessitate PFT and lung imaging.

## Abbreviations

AIDS: acquired immune deficiency syndrome; COPD: chronic obstructive pulmonary disease; CT: computed tomography; D_L, CO_: diffusing capacity of the lung for carbon monoxide; FEV_1_: forced expiratory volume in one second; FVC: forced vital capacity; HAART: Highly active antiretroviral therapy; HIV: Human immunodeficiency virus; K_CO_: Transfer coefficient; PCP: Pneumocystis carinii pneumonia; PFT: Pulmonary function tests; T_L, CO_: carbon monoxide transfer factor

## Competing interests

The authors declare that they have no competing interests.

## Authors' contributions

All authors contributed to the literature analysis, writing and final validation of the manuscript. All authors read and approved the final manuscript.
